# Realization of III–V Semiconductor Periodic Nanostructures by Laser Direct Writing Technique

**DOI:** 10.1186/s11671-016-1780-3

**Published:** 2017-01-05

**Authors:** Yuan-qing Huang, Rong Huang, Qing-lu Liu, Chang-cheng Zheng, Ji-qiang Ning, Yong Peng, Zi-yang Zhang

**Affiliations:** 1 School of Physical Sciences and Technology, Lanzhou University, 730000 Lanzhou, People’s Republic of China; 2 Key Laboratory of Nanodevices and Applications, Suzhou Institute of Nano-Tech and Nano-Bionics, Chinese Academy of Sciences, 215123 Suzhou, People’s Republic of China; 3 The NanoX, Suzhou Institute of Nano-Tech and Nano-Bionics, Chinese Academy of Sciences, 215123 Suzhou, People’s Republic of China; 4 Department of Mathematical Sciences, Mathematics and Physics Centre, Xi’an Jiaotong-Liverpool University, 215123 Suzhou, People’s Republic of China

**Keywords:** Laser direct writing, Periodic nanostructures, III–V semiconductor, Phase change materials

## Abstract

In this paper, we demonstrated the fabrication of one-dimensional (1D) and two-dimensional (2D) periodic nanostructures on III–V GaAs substrates utilizing laser direct writing (LDW) technique. Metal thin films (Ti) and phase change materials (Ge_2_Sb_2_Te_5_ (GST) and Ge_2_Sb_1.8_Bi_0.2_Te_5_ (GSBT)) were chosen as photoresists to achieve small feature sizes of semiconductor nanostructures. A minimum feature size of about 50 nm about a quarter of the optical diffraction limit was obtained on the photoresists, and 1D III–V semiconductor nanolines with a minimum width of 150 nm were successfully acquired on the GaAs substrate which was smaller than the best results acquired on Si substrate ever reported. 2D nanosquare holes were fabricated as well by using Ti thin film as the photoresist, with a side width of about 200 nm, but the square holes changed to a rectangle shape when GST or GSBT was employed as the photoresist, which mainly resulted from the interaction of two cross-temperature fields induced by two scanning laser beams. The interacting mechanism of different photoresists in preparing periodic nanostructures with the LDW technique was discussed in detail.

## Background

Semiconductor periodic structures with feature size smaller than hundreds of nanometers exhibit many unique optical and electronic properties, attracting great interests in the research fields of optoelectronics, magnetoelectronics, and bioengineering, and are highly desirable for applications in industries [1, 2]. Periodic semiconductor nanostructures have been widely used to fabricate nanophotonic devices, such as photonic crystals [2, 3] and plasmonic structures [4, 5], to acquire well-controlled light propagation in nanoscale [6], which are the crucial for applications in quantum computing and quantum communication. Site-controlled quantum dots grown on periodic nanoscaled patterns have been studied for luminescence modulation [7, 8], and nanoscale-integrated structures have also attracted much interest in fabricating spin-controlled electronic devices such as magnetic-random access memory and spin modulators [9, 10].

Electron beam lithography (EBL), deep ultraviolet lithography, interference lithography, scanning probe microscope (SPM) lithography, and nanoimprint lithography (NIL) are well-known techniques to fabricate nanometer and submicron feature structures [11–15], but expensive equipment is required for EBL, deep ultraviolet lithography, and interference lithography and the serial write mechanism of SPM lithography and EBL makes large-area patterning costly and time-consuming [16]. Although nanoimprint lithography is relatively low cost compared to those mentioned above, the initial submicron patterning technique required to fabricate a master mold or masking pattern is still an essential process [16]. Recently, the laser direct writing (LDW) technique has been proven to be a very effective and low-cost method to fabricate large-area periodic submicron structures, which demands neither a vacuum environment nor a particular light source offering a large freedom of choice of photoresists. Generally, the photoresists used in LDW are the metal film or alloy film, rather than traditional organic photoresists, which will experience structure change caused by the effect of thermochemistry or phase change due to laser action [17, 18]. Metal Ti thin film has been employed as the photoresist for LDW owing to their active thermochemistry effect and high selective etching rate between exposed and unexposed materials [18, 19]. Metal Ti changes into the TiO_2_ by laser writing, and Ti thin films can be easily removed in HF solution, leaving the desired TiO_2_ patterns [19]. Apart from acting as the mask material, TiO_2_ has many other important functions in a wide range of applications, such as high refractive index component of multilayer optical filter, antireflective coating, and planar waveguides [20]. In addition, some inorganic phase change materials (PCMs) have also been used as photoresists for LDW owing to their processable features in vacuum, photothermal response, long shelf life, and sharper boundaries between exposed and unexposed areas [21, 22]. This kind of photoresists can be easily deposited onto both planar and non-planar substrates in vacuum, greatly simplifying the process procedure by completely eliminating pre-baking and post-baking steps required for traditional organic photoresists. Feature with high speed of phase transformation and a high degree of cyclability without any compositional change between different phases, Ge_2_Sb_2_Te_5_ (GST) and Ge_2_Sb_1.8_Bi_0.2_Te_5_ (GSBT), have been used as very promising candidates as photoresists for LDW techniques [22]. Recently, many kinds of periodic patterns on Si substrate have been realized by LDW using GST and GSBT as photoresists [23–25], but there is no reported research on III–V semiconductor substrates yet.

In this paper, one-dimensional (1D) and two-dimensional (2D) periodic structures of hundreds of nanometers had been fabricated on III–V GaAs substrate utilizing the LDW technique with a nanosecond pulse laser source. Ti, GST, and GSBT films were chosen as photoresists to obtain small feature sizes. Wet-chemical etching processes were applied for development and patterns transfer to semiconductors. 1D nanolines with feature size of about 50 to 400 nm were obtained, and 2D nanoscaled square holes were prepared as well. However, the square holes could only be acquired by using Ti thin film as the photoresist, and the designed square holes changed into rectangle shape by using GST and GSBT as the photoresists mainly due to the interaction of the scanning laser beams. The effects on different photoresists of tetramethylammonium hydroxide (TMAH) and HF, acting as developing solution, and KOH and NH_4_OH as the solution for patterns transfer to semiconductors had also been widely studied and developed.

## Methods

**Fig. 1:**
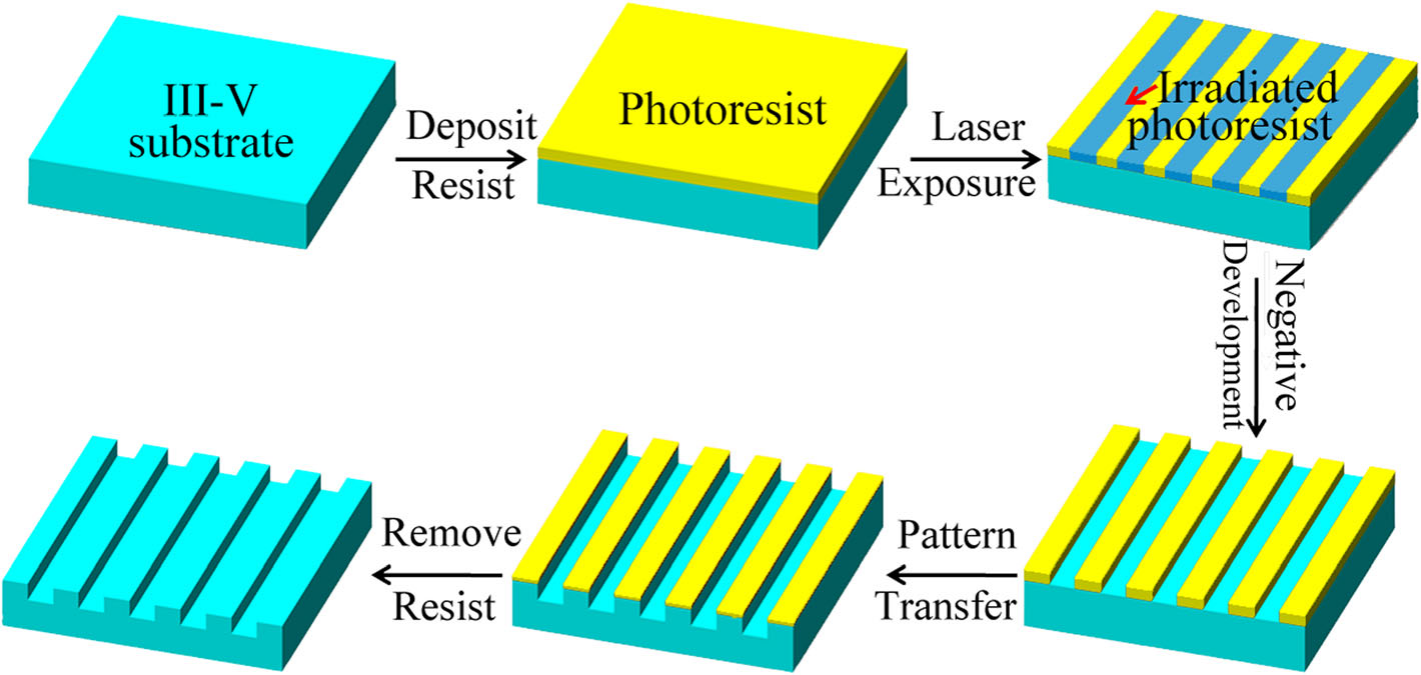
The schematic diagram of the fabrication process of nanopatterns on III–V semiconductor substrates

The schematic experimental process was shown in Fig. 1. Firstly, Ti, GST, or GSBT film was deposited onto GaAs substrate as the photoresist. Before the deposition of Ti film, a 10-nm SiO_2_ sacrificial layer was deposited onto the GaAs substrate, while GST and GSBT films were directly deposited without any underneath layers. Ti thin film was deposited by electron beam evaporation, SiO_2_ by inductive coupling plasma-assisted chemical vapor deposition (ICP-CVD) on an Oxford Plasmalab 380, and GST and GSBT films by RF magnetron sputtering on a Krut J.Lesker PVD 75. After the deposition of photoresists, lithography was performed by using LDW system (HWN LDW-P1500, laser wavelength 405 nm and 0.9NA objective lens). The samples with Ti patterns were developed in HF solution, and the samples with GST and GSBT patterns were developed in TMAH solution. Then, the developed nanolines and square nanoholes were transferred to the GaAs substrates by using (NH_4_OH and H_2_O_2_) or (KOH and H_2_O_2_) solution. The residual photoresists were volatilized by thermal annealing process for the samples with GST and GSBT patterns. The thickness of those films and the depth of the structures were analyzed by a Dektak 150 stylus profiler, the surface morphologies were characterized by atomic force microscope (AFM) on a Veeco Dimension 3100, and the phase and crystalline properties were studied with X-ray diffractometer (XRD) on a Bruker AXS D8 Advance.

## Results and Discussion

To fabricate nanoscaled periodic lines, GSBT film of 80 nm, GST film of 80 nm, and Ti film of 40 nm were deposited on GaAs substrates. With the irradiation of a focused laser beam, the local area of the film was quickly heated up to a high temperature and then rapidly cooled down to room temperature when the laser beam moved away. This process resulted in obvious structural transformation depending on the variation of volume and the surface height of photoresists [26].

**Fig. 2:**
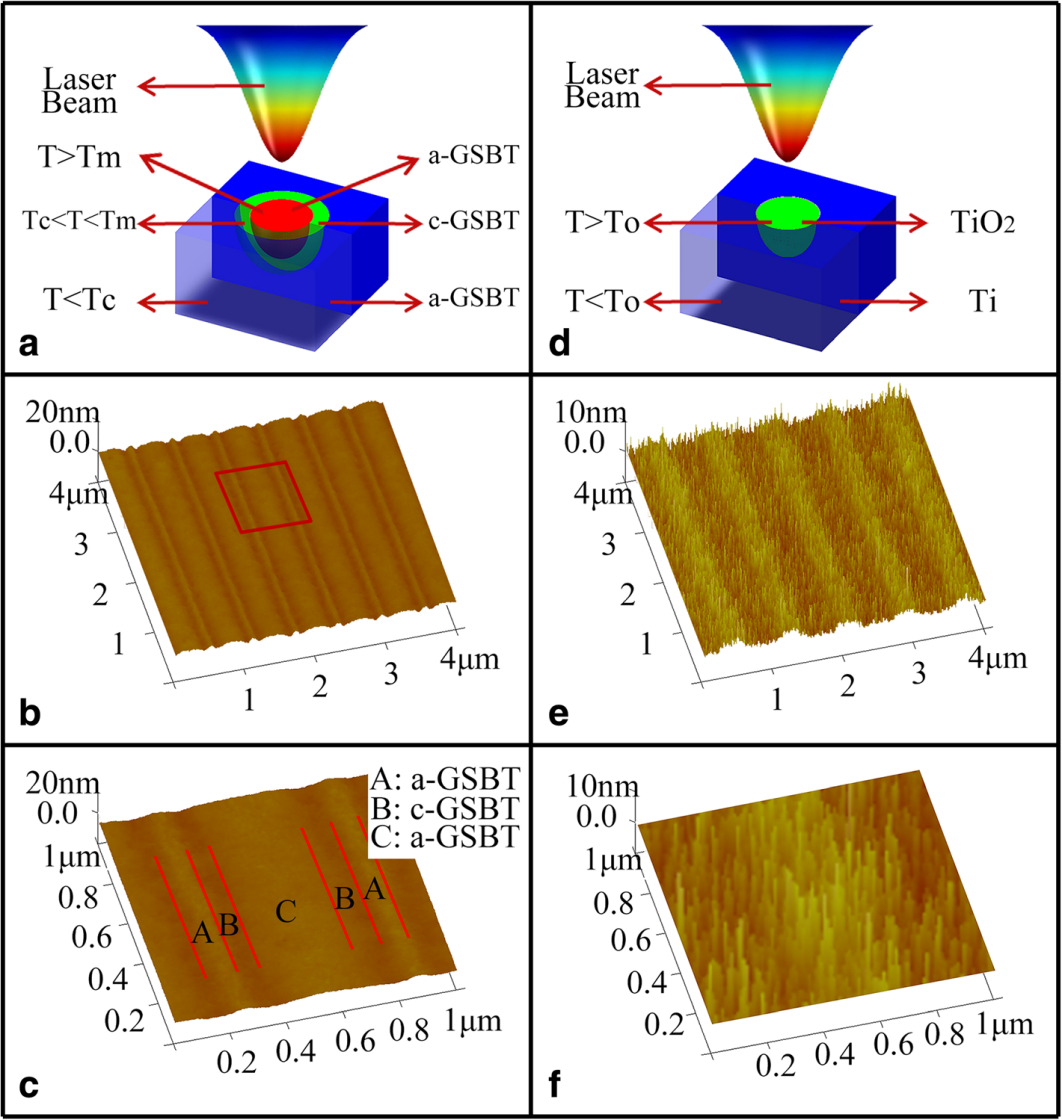
**a**, **b** The schematic diagram of the mechanism of laser action on GSBT film and the surface morphology of the GSBT film after laser irradiation. **d**, **e** The schematic diagram of the mechanism of laser action on Ti film and the surface morphology of the Ti film after laser irradiation. **c**, **f** The enlarged views of detailed structures of (**b**) and (**e**), respectively

The GSBT films irradiated by laser beam (*P* = 20 mW, duration = 50 ns, and scan rate about 3 cm/s) with a period of 600 nm were depicted in Fig. 2b, c. It is obvious that the irradiated GSBT film could be divided into three parts A, B, C, corresponding to amorphous GSBT (a-GSBT), crystalline GSBT (c-GSBT), and thin bilayer film containing a-GSBT on the surface with c-GSBT below, respectively [27]. This distribution of GSBT phases was consistent with the schematic diagram of the mechanism of laser action on GSBT film as shown in Fig. 2a, which the GSBT film was divided into three parts according to three temperature ranges induced by laser irradiation, defined by T < crystallization temperature (Tc), Tc < T < melt temperature (Tm), and T > Tm. The as-grown GSBT film is of amorphous state, which will change into the crystalline state when heated up to Tc. As shown in part B in Fig. 2c, this usually results in the volume shrinkage and the decrease of the local thickness of the film due to the larger density of the crystalline state than of the amorphous state. However, when the temperature heated by laser irradiation exceeds Tm, the surface of some parts of c-GSBT film will melt and a-GSBT is created above the underlying layer that still remains as c-GSBT. As a consequence, it was found that the surface height of part C is over than that of part B but still lower than that of part A (unexposed area), as shown in Fig. 2c. During the LDW experiment, if the maximum temperature induced by laser irradiation is always below Tm, part C will never appear and the situation will be similar to the LDW process by using Ti film as photoresist as shown in Fig. 2d–f. Compared with GSBT, the melt temperature of metal Ti is very high of 1668 °C, which is difficult for laser irradiation to reach, so that the heating effect on Ti film will always maintain the temperature below Tm in the whole experiment. During the laser writing process, Ti film will be changed into titanium oxide (TiO_2_) when it was heated above the oxidation temperature (To). The oxidation of Ti is usually accompanied with an increased volume [19]. Although, as shown in Fig. 2e, f, the apparent contrast between the exposed area and unexposed area of Ti film indicates the appropriate exposure laser power has been used, the much rough surface of the sample compared with that of the GSBT sample may lead to a challenge for getting small feature size in the following fabrication processes of the semiconductor nanostructures.

**Fig. 3:**
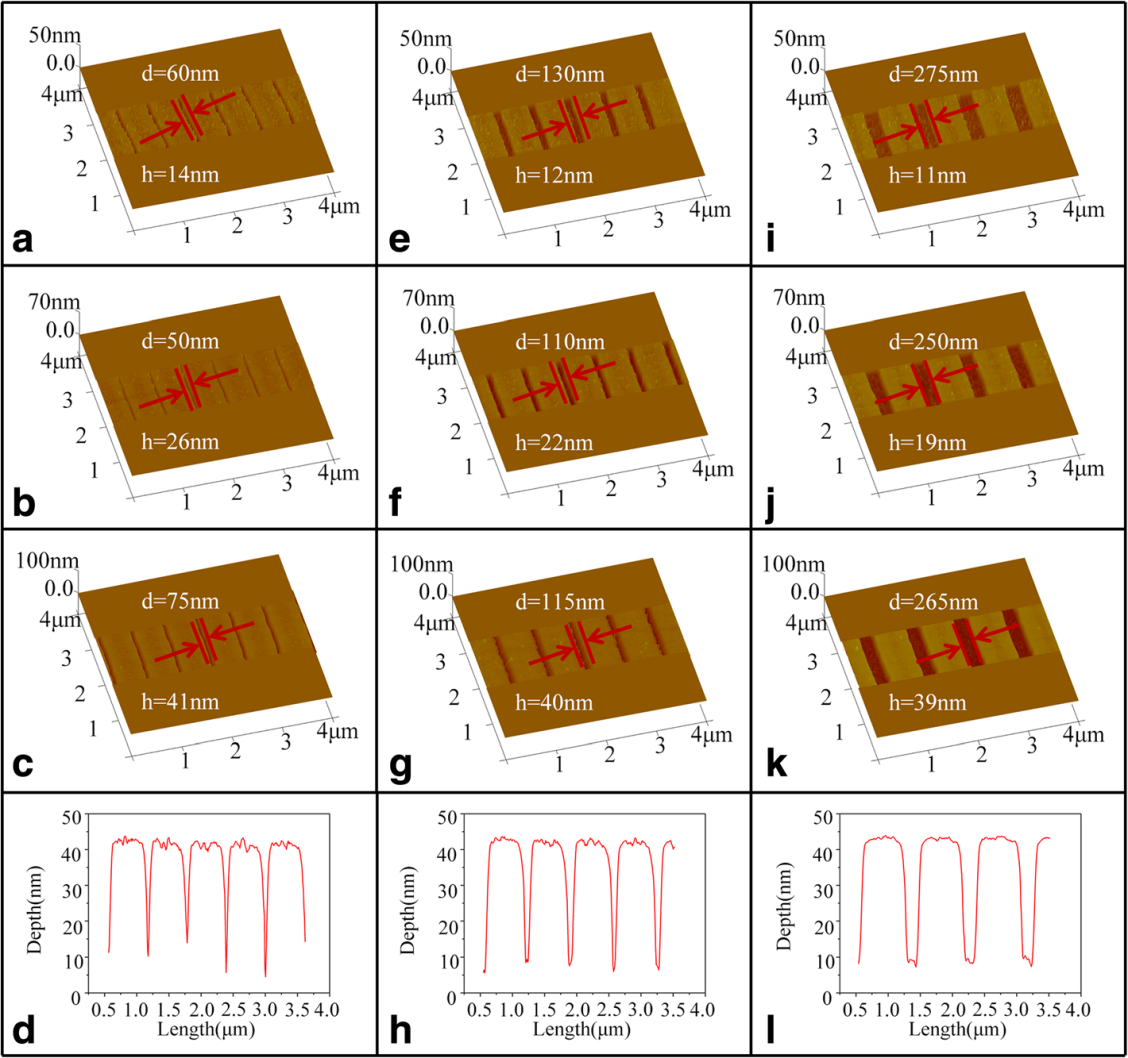
**a**–**c** AFM images of GSBT films irradiated with a period of 600 nm for 20, 40, and 80 min developments, respectively. **d** The corresponding cross-sectional profile of (**c**). **e**–**g** AFM images of GSBT films irradiated with a period of 675 nm for 20-, 40-, and 80-min developments, respectively. **h** The corresponding cross-sectional profile of (**g**). **i**–**k** AFM images of GSBT films irradiated with a period of 900 nm for 20-, 40-, and 80-min developments, respectively. **l** The corresponding cross-sectional profile of (**k**)

After laser exposure, GSBT samples which were designed with different periods (nanoline and the separation between the two adjacent nanolines) of 600, 675, and 900 nm were chosen for the following development process. A basic solution of 25% TMAH was employed to corrode a-GSBT away, and GSBT served as a negative resist in this case because the etching rate of a-GSBT is larger than that of the c-GSBT in TMAH solution. The AFM results of the GSBT samples acquired with different developing times were shown in Fig. 3. As illustrated in Fig. 3b, the nanolines with a very narrow width of about 50 nm about a quarter of the optical diffraction limit was successfully realized in the GSBT films, which is much smaller than 140 nm, the narrowest width of nanolines fabricated by the LDW technique using PCMs as photoresists ever reported by other research groups [28]. Moreover, by comparing the results in Fig. 3a–c, it was found that the width of nanolines after 20-min etching is about 60 nm but decreases to 50 nm after 40-min etching and then increases to 75 nm after etched for 80 min. This phenomenon is different from the general relation between the width of nanolines and the developing time, where the width always increases with the increase of developing time. This interesting result observed in our experiments may be attributed to two factors, the side corrosion and the apple core-like (wide-narrow-wide) a-GSBT material distribution inside the surface of the film by laser irradiation. The first factor of side corrosion always results in an increase of the width of the nanolines with longer etching time, while the apple core-like effect leads to decrease of the width before some certain time (“the upper half part of the apple core”) and then increase afterwards (“the rest part of the apple core”), the same as that of side corrosion. For the developing time shorter than 40 min, the “upper part of the apple core” of the later factor is dominated, exhibiting a decreasing trend of the widths of nanolines with time, and for longer developing time than 40 min, the widening effect dominated by the “rest part of the apple core” results in the increase of the nanolines width. Similar results were also observed as in Fig. 3e–g, i–k. Different from the relation of width with developing time, the depth of the nanolines always increases with the developing time. As shown in Fig. 3, the depths are 14, 26, and 41 nm for the sample with the period of 600 nm developed for 20, 40, and 80 min, respectively, 12, 22, and 40 nm for the sample with the period of 675 nm, and 11, 19, and 39 nm for the sample with the period of 900 nm. The surface is very smooth, and the boundary is very sharp between the exposed and unexposed area of the samples after developing process, which are further confirmed by the characterization of the cross-sectional profiles of the samples as seen in Fig. 3d, h, i. These processing results benefit the following pattern transfer process as what will be shown in the following part.

**Fig. 4:**
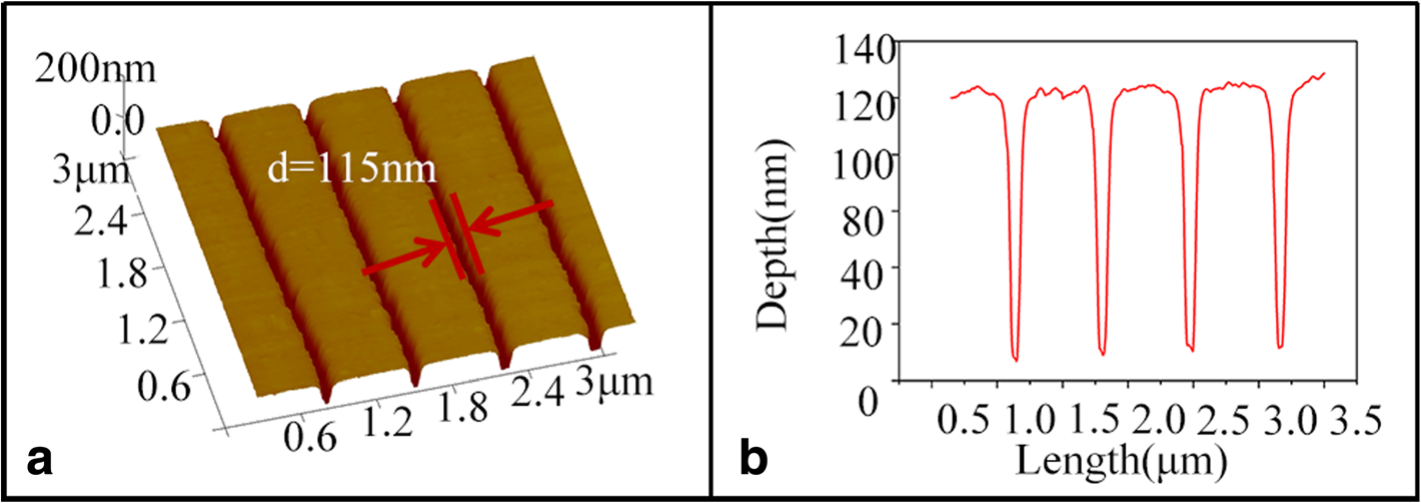
**a** The AFM image of the surface morphology of the sample with 675 nm period after pattern transfer. **b** The corresponding cross-sectional profile of the sample

The samples with line widths of 50, 75, and 115 nm were selected to transfer the GSBT pattern to the GaAs substrate. The pattern transferring process was executed by using NH_4_OH (25%):H_2_O_2_ (30%):H_2_O = 1:0.2:50 solution treated for 20 s. Among these samples, only the nanolines with the width of about 115 nm were successfully transferred to the GaAs substrate, as shown in Fig. 4a, while the others did not. This might be due to the lack of the reaction in the very narrow (50 and 75 nm) grooves of the nanolines during the selective etching process between GaAs and c-GSBT using the above etching solution. From the AFM image in Fig. 4a and the corresponding cross-sectional profile in Fig. 4b, it can be seen that the transferred semiconductor nanolines are uniform with the width and depth of about 115 and 95 nm, respectively.

**Fig. 5:**
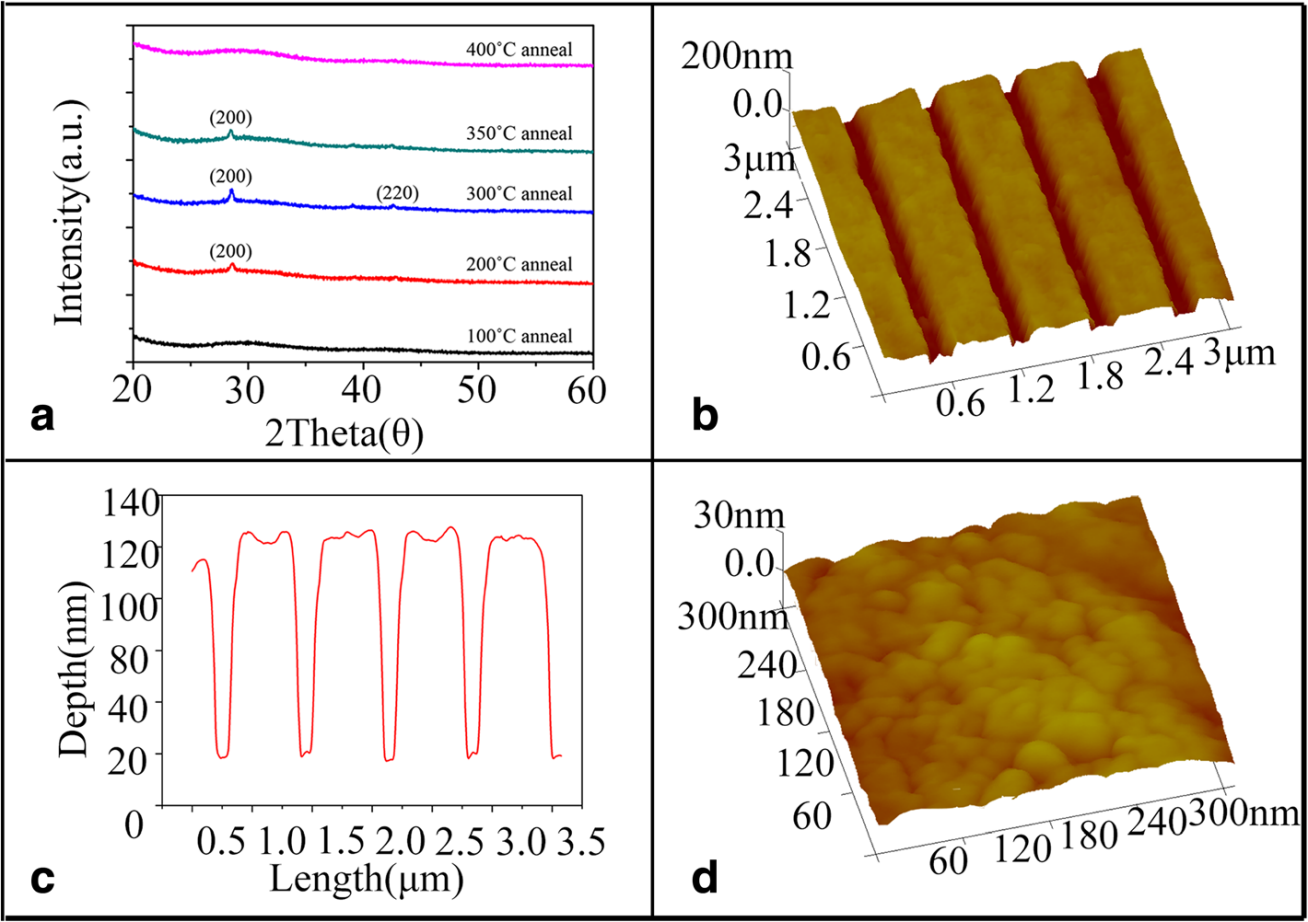
**a** The XRD result of the annealed samples. **b** The surface morphology of the annealed sample. **c** The corresponding cross-sectional profile of the annealed sample. **d** The enlarged image for detailed view of the annealed sample

It is a necessary process step to remove the residual GSBT resist after pattern transfer process. In our experiments, the residual GSBT resist was cleaned by annealing in vacuum, in which the temperature was chosen between Tg of GSBT and Tm of GaAs (1238 °C). The GSBT films were annealed at different temperatures for test. The XRD results in Fig. 5a show the phase change of GSBT on GaAs surface upon annealing treatment. The diffraction of peak indexed as (200) evidences the formation of c-GSBT at the annealing temperature of 100 °C, which is about the Tc of GSBT. The intensity of the diffraction peak (200) increases at first, then decreased with the increase of the annealing temperature, and finally vanished at the temperature of 400 °C, indicating the removal of the residual GSBT at this temperature. Based on these test results, an annealing treatment with 420 °C annealing temperature for 10 min was employed. After the annealing process, from the AFM image shown in Fig. 5b and the corresponding cross-sectional profile shown in Fig. 5c of the annealed sample, the depth and width of nanolines are measured as 84 and 150 nm, respectively, which are consistent with the result shown in Fig. 4, further indicating that the residual photoresists have been completely removed. This result is better than the previous report of 210 nm line width on SiO_2_ substrate presented by other research groups using PCMs as photoresists by LDW process [25]. And a smooth surface with the average surface roughness about 3.02 nm was obtained in the annealed sample as seen in the image of the surface morphology of the ridge of the annealed sample in Fig. 5d.

**Fig. 6:**
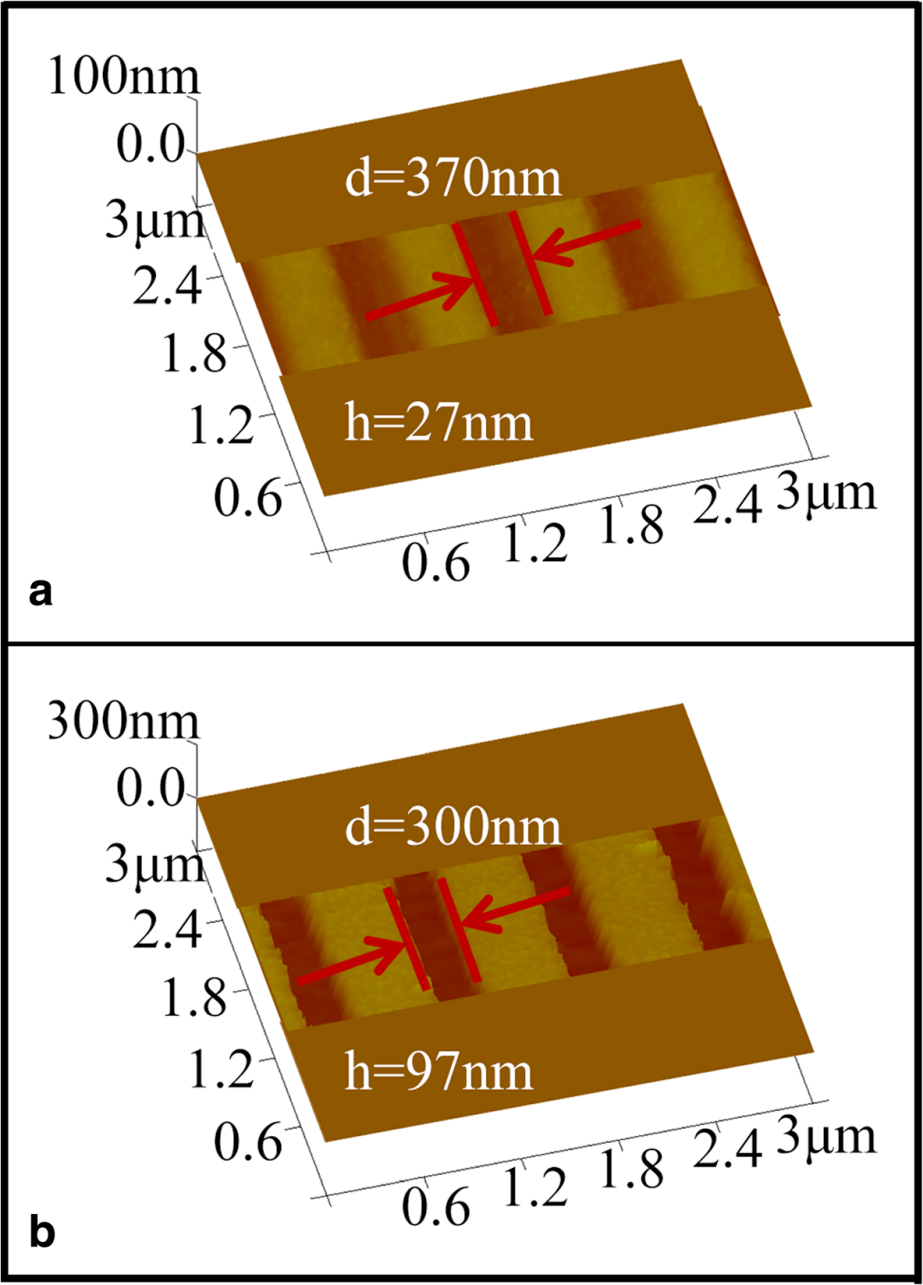
**a**, **b** The images of GST films with period of 800 nm after development and pattern transfer to semiconductors, respectively

Besides GSBT film, GST films with thickness of 80 nm were also prepared to fabricate nanolines on GaAs. The period of the GST pattern was designed as 800 nm. After laser irradiation (*P* = 30 mW, duration = 40 ns, and scan rate about 3 cm/s), the samples were developed by using basic solution (25% TMAH solution) for 630 s. Figure 6a showed the image of GST pattern after development, indicating a depth of about 27 nm and the width of about 370 nm of the nanolines on GST film. But the side walls of the nanolines are found very sloppy, which is quite different from the results of the developed GSBT films where the side walls are steep and sharp. The sharp side wall (the sharp boundary between exposed area and unexposed area) is a crucial factor for fabricating a pattern with very small feature size. The main reason for this difference between the two PCMs, GST and GSBT, is attributed to the larger Tc of GST film compared to that of GSBT. The following process of pattern transferring to GaAs substrate was executed by using KOH (48%):H_2_O_2_ (30%):H_2_O = 1:3.5:70 solution for 6 s. As seen in Fig. 6b, the depth and the width of the nanolines on GaAs substrates are 97 and 300 nm, respectively. The narrower width (300 nm) of the nanolines on GaAs than (370 nm) on GST pattern was found, which is due to the same reason as described above in Fig. 3. Moreover, as analyzed above, the sloppy side walls after development process lead to the rough and not straight patterns on semiconductor as shown in Fig. 6b, which were detrimental for the application of obtained nanostructures. As a result, among the two PCM photoresists, GSBT film has advantages for fabricating high-quality semiconductor nanostructures.

**Fig. 7:**
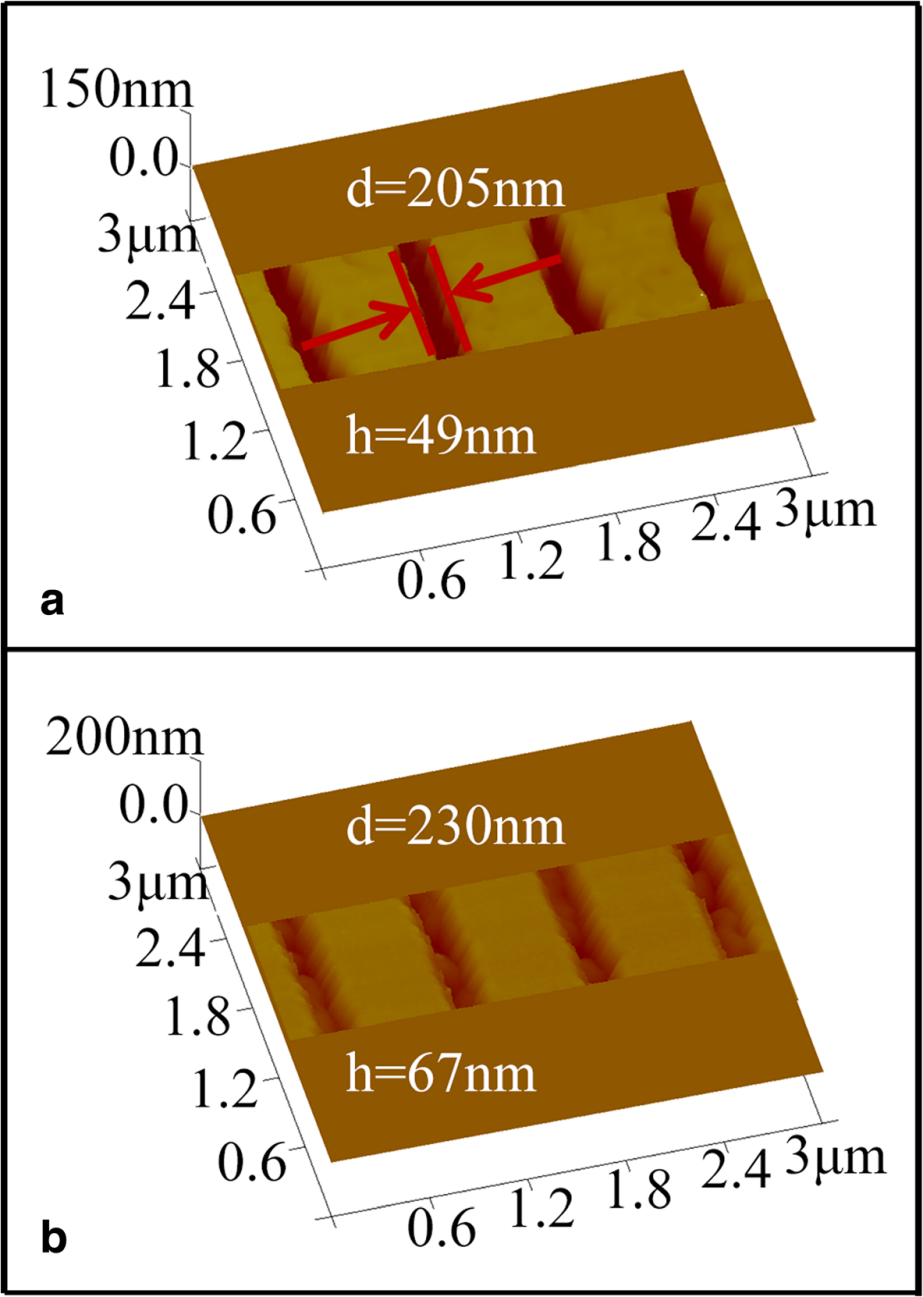
**a**, **b** The images of laser-induced materials changed Ti film with a 800-nm period after developed for 55 s and pattern transfer, respectively

Besides the experiments of LDW using GST and GSBT as the photoresists, metal Ti thin films were also prepared to fabricate the nanoline structures on GaAs substrates. With higher selective etching rate between exposed and unexposed materials compare to PCM films, it was expected that the fabricated nanolines with Ti film may realize high aspect ratio. After depositing 10 nm of SiO_2_ film onto the GaAs substrate as a buffer layer, a Ti layer of 40 nm was deposited as the photoresist by the electron beam evaporation with a speed of 0.1 nm/s. The Ti films were irradiated by the laser (*P* = 85 mW, duration = 2000 ns, and scan rate about 3 cm/s) with a period of 800 nm and then developed by dipping the samples in the HF (40%):H_2_O = 1:100 solution for 55 s. As shown in the Fig. 7a, the width of the obtained nanolines was about 205 nm, with a depth of 49 nm after development, and the side walls appear very rough, looking like sawtooth. As mentioned above, the irradiated areas were changed into TiO_2_ which are very difficult to be removed, while the unirradiated areas can be removed easily in this process indicating the very high etching selectivity. We believe that the corrosion resistant performance of TiO_2_ is the main reason for the rough side walls of the nanolines, because of a large monocrystals of TiO_2_ created in the Ti film due to laser irradiation. The nanolines developed in Ti thin films were then transferred to the GaAs substrates by using KOH (48%):H_2_O_2_ (30%):H_2_O = 1:3.5:70 solution treated for about 6 s. The width of the nanolines (Fig. 7b) gets about 230 nm with depth of about 67 nm after pattern transfer. Compared with the results of GSBT films, Ti film exhibits some unexpected disadvantageous effects such as the rough boundary and the rough ravine, which had serious influence on the morphology of the patterned semiconductor nanostructures.

**Fig. 8:**
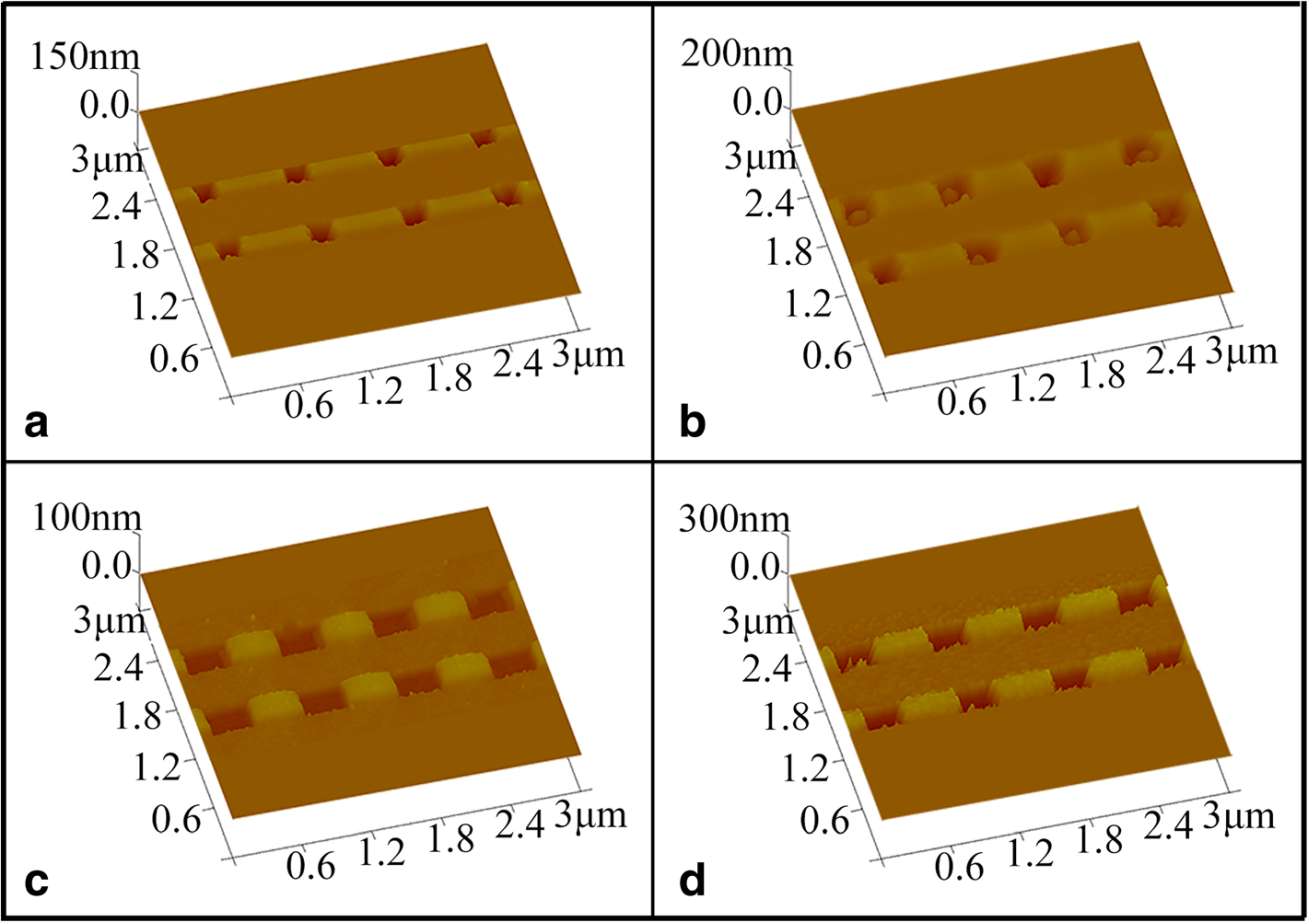
**a**, **b** The 2D nanosquared images of laser-induced Ti film with 800-nm period after development and pattern transfer, respectively. **c**, **d** The 2D nanosquared images of laser-induced phase changed GST film with 800-nm period after development and pattern transfer, respectively

2D square nanoholes were also prepared by using Ti and GST as the photoresists on the GaAs substrates. The laser irradiation parameters for Ti films are 85 mW irradiation power, 2000 ns duration, and 3 cm/s scan rate and for GST films are 30 mW irradiation power, 40 ns duration, and 3 cm/s scan rate. As seen in Fig. 8a, the squared holes with a line width of about 200 nm and a depth of about 50 nm were successfully realized on Ti films. After the pattern transferred to GaAs substrates, the depth and line width of the square holes (in Fig. 8b) were changed into 69 and 220 nm, respectively, with obvious trapezial distortion and rough boundary, as what was observed with the 1D nanoline structures as described in Fig. 7. In the case of GST films, the developed nanoholes (shown in Fig. 8c) appeared distorted to a rectangle shape with 27 nm in depth, 330 nm in length, and 220 nm in width, which become 110, 350, and 240 nm, respectively, after transferred to the GaAs substrate. The distortion of the nanopatterns by using GST as photoresist is mainly caused by the interaction of the two temperature fields induced by cross-scanning laser beams during the LDW process. The interaction of two temperature fields is similar to that of two electric fields, and the distribution of temperature field is similar to the intensity distribution of an electric field, which leads to the overhead view of the temperature field and the phase change area looking like a waxing gibbous moon. And hence, less influence is expected in scan direction than its normal direction. After the exposure to laser irradiation, the area of a-GST turned to a rectangle shape and therefore reproduced rectangle holes after transferring process. Since Ti changes into TiO_2_ at a high temperature, the increased temperature induced by laser irradiation might only increase the reaction rate slightly, making Ti film much less sensitive to the heating effect than GST film.

## Conclusions

In summary, 1D nanolines with the line width of 50 nm quarter of the optical diffraction limit were obtained on GSBT films, and 150 nm in line width on GaAs substrates, which are much smaller than the best results acquired on Si substrate ever reported. 2D nanoholes have also been fabricated, indicating the effect of two temperature fields induced by scanning laser beams which can decide the final shape of the 2D patterns. This work demonstrates very attractive capabilities of LDW to fabricate 1D and 2D III–V semiconductor nanostructures, revealing great potential for applications in bioengineering and optoelectronics fields. Further studies are required in the future work to minimize the feature size of semiconductor nanostructures by improving LDW process via dry etching instead of wetting etching to reduce side-corrosion effects.

## Abbreviations

1D: One dimension
2D: Two dimension
AFM: Atomic force microscope
a-GSBT: Amorphous GSBT
c-GSBT: Crystalline GSBT
EBL: Electron beam lithography
GSBT: Ge_2_Sb_1.8_Bi_0.2_Te_5_
GST: Ge_2_Sb_2_Te_5_
ICP-CVD: Inductive coupling plasma-assisted chemical vapor deposition
LDW: Laser direct writing
NIL: Nanoimprint lithography
PCM: Phase change material
SPM: Scanning probe microscope
Tc: Crystallization temperature
Tm: Melt temperature
TMAH: Tetramethylammonium hydroxide
To: Oxidation temperature
XRD: X-ray diffractometry
